# Use of facility assessment data to improve reproductive health service delivery in the Democratic Republic of the Congo

**DOI:** 10.1186/1752-1505-3-12

**Published:** 2009-12-21

**Authors:** Sara E Casey, Kathleen T Mitchell, Immaculée Mulamba Amisi, Martin Migombano Haliza, Blandine Aveledi, Prince Kalenga, Judy Austin

**Affiliations:** 1 RAISE Initiative, Heilbrunn Department of Population and Family Health, Mailman School of Public Health, Columbia University, New York, USA; 2 International Rescue Committee, New York, USA; 3 International Rescue Committee, Bukavu, Democratic Republic of the Congo; 4 International Rescue Committee, Kisangani, Democratic Republic of the Congo; 5 International Rescue Committee, Kinshasa, Democratic Republic of the Congo; 6 CARE, Kasongo, Democratic Republic of Congo

## Abstract

**Background:**

Prolonged exposure to war has severely impacted the provision of health services in the Democratic Republic of the Congo (DRC). Health infrastructure has been destroyed, health workers have fled and government support to health care services has been made difficult by ongoing conflict. Poor reproductive health (RH) indicators illustrate the effect that the prolonged crisis in DRC has had on the on the reproductive health (RH) of Congolese women. In 2007, with support from the RAISE Initiative, the International Rescue Committee (IRC) and CARE conducted baseline assessments of public hospitals to evaluate their capacities to meet the RH needs of the local populations and to determine availability, utilization and quality of RH services including emergency obstetric care (EmOC) and family planning (FP).

**Methods:**

Data were collected from facility assessments at nine general referral hospitals in five provinces in the DRC during March, April and November 2007. Interviews, observation and clinical record review were used to assess the general infrastructure, EmOC and FP services provided, and the infection prevention environment in each of the facilities.

**Results:**

None of the nine hospitals met the criteria for classification as an EmOC facility (either basic or comprehensive). Most facilities lacked any FP services. Shortage of trained staff, essential supplies and medicines and poor infection prevention practices were consistently documented. All facilities had poor systems for routine monitoring of RH services, especially with regard to EmOC.

**Conclusions:**

Women's lives can be saved and their well-being improved with functioning RH services. As the DRC stabilizes, IRC and CARE in partnership with the local Ministry of Health and other service provision partners are improving RH services by: 1) providing necessary equipment and renovations to health facilities; 2) improving supply management systems; 3) providing comprehensive competency-based training for health providers in RH and infection prevention; 4) improving referral systems to the hospitals; 5) advocating for changes in national RH policies and protocols; and 6) providing technical assistance for monitoring and evaluation of key RH indicators. Together, these initiatives will improve the quality and accessibility of RH services in the DRC - services which are urgently needed and to which Congolese women are entitled by international human rights law.

## Background

### Reproductive Health among Conflict-Affected Populations

Complex humanitarian emergencies caused by armed conflict are characterized by social disruption, population displacement and collapse of public health infrastructure [1]. Humanitarian assistance for refugees and internally displaced persons (IDPs) requires specific attention to ensure that the reproductive health (RH) rights of the population are recognized. Women living in conflict and post-conflict settings are faced with many RH concerns including high risk of death or disability due to pregnancy-related causes, lack of information about and access to family planning (FP), complications following unsafe abortion, gender-based violence and sexually transmitted infections (STIs) including HIV [2]. Women and men affected by armed conflict have the right to RH-related information and access to safe, effective, affordable and acceptable FP methods as well as appropriate and effective health care services that will enable women to experience safe pregnancies and childbirth [3].

The *Minimum Initial Services Package (MISP) for RH in Crisis Situations *establishes a set of priority RH interventions to be implemented during the earliest days of a humanitarian crisis and calls for early planning for the introduction of comprehensive RH services once the emergency situation has stabilized. Although implementation of the MISP is a standard in the Sphere Project's *Humanitarian Charter and Minimum Standards in Disaster Response *[4], emergency obstetric care (EmOC) and FP services are still rarely available to populations affected by armed conflict [2,5].

The largest public health disparity between developed and developing countries is maternal mortality [6]. Progress towards achieving the fifth Millennium Development Goal (MDG) to reduce maternal mortality by three-quarters by 2015 is inadequate [7]. Evidence suggests that conflict-affected countries are even further from achieving this goal [8]; nine of the ten lowest-ranked countries in Save the Children's *2009 Mothers' Index *are either currently affected by armed conflict or emerging from recent conflict [9].

Most maternal deaths in developing countries result from direct obstetric complications, which include hemorrhage, sepsis, pre-eclampsia and eclampsia, prolonged or obstructed labor and complications of abortion [10,11]. Most direct obstetric complications are treatable and maternal deaths may be avoided if complications are treated properly and in time. The majority of maternal deaths occur during labor, delivery or the first 24 hours postpartum and many can be attributed to at least one of the "three delays" that occur before a woman receives a life-saving intervention: delay in deciding to seek care on the part of the woman and/or her family; delay in reaching a facility that provides EmOC; and delay in receiving good quality care at the facility [12]. Since most obstetric complications cannot be predicted or prevented [6], access to safe and effective obstetric services, including EmOC, is crucial to averting maternal morbidity and mortality.

Nine signal functions, or life-saving interventions that are used to treat direct obstetric complications, are used to monitor and assess a health facility's capacity to provide EmOC services and avert maternal deaths [10,13]. The guidelines for monitoring EmOC services were recently updated to include an additional signal function on treatment of complications in newborns [13]. A health facility is defined as a basic EmOC facility if the following services, which can be performed at health center level, are available 24 hours a day/7 days a week and have been performed in the past three months:

1. Administer parenteral antibiotics,

2. Administer uterotonic drugs (e.g. parenteral oxytocin),

3. Administer parenteral anticonvulsants for pre-eclampsia and eclampsia,

4. Manually remove the placenta,

5. Remove retained products of conception,

6. Perform assisted vaginal delivery and

7. Perform basic neonatal resuscitation (e.g. with bag and mask) [10,13,14].

To be classified as a comprehensive EmOC facility, all of the above criteria must be met and the following two signal functions must have been performed at least once in the preceding three months:

8. Perform surgery (e.g. cesarean delivery) and

9. Perform blood transfusion [10,13].

The lack of infection prevention practices in the health facility environment is a common barrier to providing good quality RH services. Sepsis is one of the leading causes of maternal deaths in developing countries and is the second leading cause of maternal deaths in the Democratic Republic of the Congo (DRC) [15]; it is also one of the most preventable of all postpartum morbidities [6,16]. Infection prevention practices in the hospital setting, including disinfection and sterilization of surfaces and equipment in the delivery room and operating theatre, use of protective devices such as aprons and gloves and use of aseptic techniques before and during delivery (cleansing with soap, disinfectant, chlorine bleach), can reduce the risk of infection to mothers and newborns [16,17]. In addition, treatment of antepartum infections plays an important role in reducing postpartum infections [16].

Family planning provides women and men with the opportunity to enjoy their reproductive rights and plays an important role in reducing maternal mortality through the prevention of unwanted pregnancy and unsafe abortion. FP also contributes to a reduction in both maternal and infant mortality by changing the structure of childbearing (age and parity of pregnant women and the time between pregnancies) [3,18]. Evidence regarding fertility preferences among conflict-affected populations is mixed, with studies showing both a desire to replace lost family members and a reluctance to become pregnant in the unstable conditions of war [19]. Despite this variance, the demand for FP services exists among nearly all conflict-affected populations, making the availability of good quality FP services critical [19]. A global evaluation of RH services in conflict-affected settings found that although 90% of sites had at least one FP method available, only half of the sites reported offering long-term methods such as the intra-uterine device (IUD) and one-third reported that sterilization was available [5].

### Reproductive Health in the Democratic Republic of the Congo

After nearly a decade of civil war, the DRC remains in the midst of a complex humanitarian emergency, faced with a devastated health infrastructure, high mortality rates and a disrupted civil society. Between 1998 and 2004, an estimated 3.9 million excess deaths occurred as a result of the conflict in the DRC [20]; the crude mortality rate in 2006 was more than 70% higher than that reported in the 1984 census [21]. The effects of the conflict on women's health and well-being are profound, with the country ranking as the worst conflict zone in the world in which to be a woman or child [22].

The destruction of the health infrastructure during the war has led to insufficient capacity to meet the health needs of the population [23] and has resulted in poor RH status and avoidable deaths. The maternal mortality ratio in the DRC is estimated to be between 549 and 1100 maternal deaths per 100,000 live births, among the highest in the world [24,25]. The major causes of maternal mortality in the DRC are hemorrhage (25%), sepsis (15%), eclampsia/pre-eclampsia (13%) and unsafe abortion (13%) [15]. Contraceptive prevalence is very low, with 6.7% of women of reproductive age reporting current use of a modern method of FP, ranging from 3.3% in rural areas to 9.5% in urban areas [24].

Also underlying the poor health infrastructure are the poor compensation and motivation of currently-employed health workers and the deterioration of the health worker education system. Many health workers have not been paid for decades and many rural health workers migrate to the cities or go to work for international agencies to seek paid employment with a regular salary [23]. In addition, little commitment to continuing professional development and in-service training is evident in the public sector, unless specifically funded by an external donor. Few health workers receive continuing medical education or training on updated medical practices or are exposed to updated medical information, such as journal articles.

Although many of the same problems can be found in other developing countries, evidence suggests that the long-term damage that armed conflict causes to health systems and to the health status of the population persists well after the conflict has ended and that women and children are disproportionately affected [26].

### The RAISE Initiative in the DRC

The Reproductive Health Access, Information and Services in Emergencies (RAISE) Initiative collaborates with partner agencies to bring together the tools needed to make comprehensive RH care in emergencies a basic standard of care. Established by the Heilbrunn Department of Population and Family Health in the Columbia University Mailman School of Public Health and Marie Stopes International, the RAISE Initiative aims to address the full range of RH needs for refugees and IDPs by building partnerships with humanitarian and development agencies, governments, United Nations (UN) bodies, advocacy agencies and academic institutions [2]. In 2007, the RAISE Initiative began working with the International Rescue Committee (IRC) and CARE in the DRC to ensure that good quality comprehensive facility-based RH care would be available to conflict-affected populations. Facility assessments were conducted to evaluate the existing capacities of health facilities to meet the RH needs of the population and to determine the availability, utilization and quality of RH services, including EmOC and FP, at supported facilities.

### Facility Assessment

A functioning health infrastructure at the facility level is crucial to the delivery of RH services. It is important, therefore, to measure facility level data [27]. Research has shown that in order to reduce maternal mortality, three facility level components need to be in place: application of good quality medical technology and use of skilled clinical providers; good management and organization within the facility including personnel, equipment, drugs and supplies; and a respect for human rights efforts to improve functioning at the facility level [28,29].

**Figure 1:**
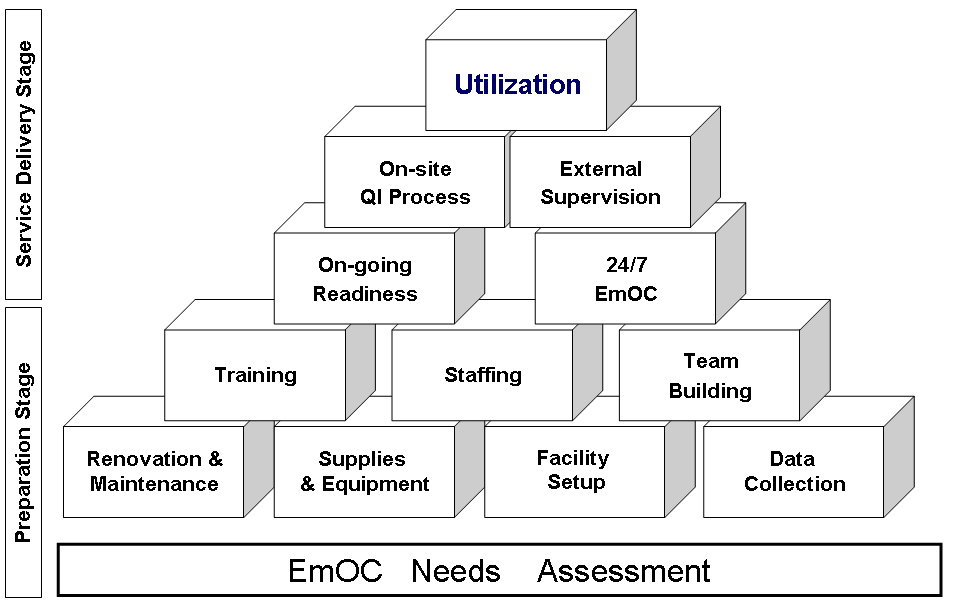
**AMDD EmOC Building Blocks Framework**.

The Averting Maternal Death and Disability (AMDD) Program's EmOC Building Blocks Framework (Figure 1) includes a plan for upgrading a facility from initial preparation through increasing utilization of services. This framework serves as a blueprint for organizing program priorities and has also proved useful in conflict settings [29,30]. As a first step in the process of improving RH care, it is important to have a clear understanding of the service delivery environment. Facility assessments provide crucial information about the content and delivery of RH services and can also identify gaps and obstacles to providing those services [31] both prior to and during program implementation [32-34]. They provide useful evidence from which to make concrete recommendations to governments and other key stakeholders for improving policies and structures.

## Methods

In 2007, RAISE, IRC and CARE conducted baseline RH facility assessments of nine general referral hospitals in nine health zones in five provinces of the DRC. The assessments served to provide baseline data for the projects and to guide project planning and implementation. Data collection teams made up of IRC, CARE and Ministry of Health (MOH) staff were trained with technical assistance from RAISE [35]. The facility assessments were conducted in March and April 2007 in South Kivu, Orientale and Kasai Occidental provinces and in November 2007 in Ndjili/Kinshasa and Maniema provinces. All nine hospitals were in areas affected by the conflict, with those in Maniema, Orientale and South Kivu particularly hard hit during the war. Periodic insecurity and population displacement continued to affect the health zones in Maniema and South Kivu at the time of the assessments.

The assessments evaluated the facilities' general infrastructure, human resources, obstetric services including EmOC, FP services and infection prevention environment. The data collection tool was adapted from the AMDD Program EmOC Facility Assessment tool [36] to include data on FP, STIs and clinical response to sexual assault, and was translated into French. The assessments incorporated multiple data collection methods including interviews with facility staff, observation and clinical records review to provide a snapshot of the services available on the day of the assessment. A room-by-room walkthrough and inventory of essential drugs, supplies and equipment were used to assess the readiness of the facility to respond to an obstetric emergency [29]. Equipment and supplies were noted as available only if they were functioning and located in the specific room where they would be used and therefore immediately available in the event of an emergency. Drugs were only noted as available for emergency use if they were present in the treatment room or facility pharmacy and were unexpired.

General infrastructure was assessed in terms of the availability of power and water sources, transportation for emergencies as well as the total number of in-patient beds and the number of designated maternity beds in the hospital. FP services were measured by the ability of the facility to provide counseling, daily oral contraceptive pills, injectables, IUDs, hormonal implants, male and female condoms, vasectomy, tubal ligation and emergency contraception. Infection prevention practices were documented in the delivery room, maternity ward, operating theatre, laboratory and the outpatient consultation room. Data from the 12 months prior to the assessment were collected on obstetric complications treated, signal functions performed, maternal deaths and FP utilization at each facility.

To assess the level of care that each facility was providing on the day of the assessment, data for each EmOC signal function were collected on whether the functions were performed in the preceding three months at the facility (as reported by maternity staff) and their availability 24 hours a day/seven days a week. The checklist that was used to determine if the facility had the essential package of staff, equipment, drugs and supplies to perform each signal function was developed from recommendations in two publications: *Managing complications in pregnancy and childbirth: A guide for midwives and doctors *[37] and *Essential Medicines for Reproductive Health *[38]. For a facility to be designated capable of providing a particular service, the full package of essential equipment, supplies, drugs and staff necessary to perform the signal function or provide the FP method must have been in evidence on the day of the assessment. Although neonatal resuscitation was not considered a signal function at the time of the assessment, data on this procedure were collected and the new signal function is included in our analysis.

When facility staff reported that they had not provided an EmOC or FP service in the preceding three months, they were asked to identify the most important reason from one of five categories: 1) *training issues *including lack of training and lack of confidence in providers' skills; 2) *supplies, equipment or drugs not being available or functional*; 3) *management issues *such as providers being encouraged to perform alternative procedures or being uncomfortable or unwilling to perform the procedure for reasons unrelated to training; 4) *policy issues *such as the required cadre of staff not being posted to the facility or national/hospital policies prohibiting the performance of the function; and 5) *lack of client demand *for the procedure during the time period under review.

Facilities were classified as having a monitoring system in place if data on obstetric complications, signal functions and FP use could be collected from registers, logs or patient files. If these data were not recorded, the facility was classified as lacking a monitoring system.

## Results

### General Infrastructure

Eight of the nine general referral hospitals assessed were government facilities while one was a Catholic hospital; four of these government hospitals were managed by religious institutions. Table 1 highlights the infrastructure of the nine general referral hospitals. The catchment area populations served by the hospitals ranged from 77,584 to 252,917. Inpatient capacity varied with the number of beds ranging from 39 to 193, of which designated maternity beds ranged from eight to 120. Six facilities had functioning power supplied by electric lines, generators and/or solar panels. All of the hospitals reported functioning water systems, with water gathered from various sources including internal and external piping, rainwater collection and delivery from external sources. Only three hospitals had a functioning designated ambulance.

**Table 1:** General Infrastructure of 9 general referral hospitals, DRC

Health zone, Province	Operating Agency	Catchment area population	Number of beds (maternity beds)	Functioning power	Source of water	Designated ambulance
**Demba**, Kasai Occidental	Government	252,917	56 (5)	No	Internal piping	No

**Mutoto**, Kasai Occidental	Government/Religious*	104,150	39 (16)	No	Rainwater collection	No

**HASC**, Ndjili	Government	249,308	137 (23)	Yes	Internal piping	Yes

**Kikimi**, Ndjili	Religious Mission	198,997	101 (38)	Yes	Internal piping	Yes

**Roi Baudouin**, Ndjili	Government	247,023	125 (20)	Yes	Internal piping	Yes

**Kasongo**, Maniema	Government/Religious*	178,821	193 (28)	Yes	Rainwater collection, external delivery	No

**Ubundu**, Orientale	Government	77,584	64 (8)	No	External delivery	No

**Kabare**, South Kivu	Government/Religious*	148,812	130 (120)	Yes	External pipes	No

**Kalehe**, South Kivu	Government/Religious*	101,136	95 (28)	Yes	Internal piping, rainwater collection	No

*Government/Religious indicates a government facility managed by a religious institution.

### Emergency Obstetric Care

**Figure 2:**
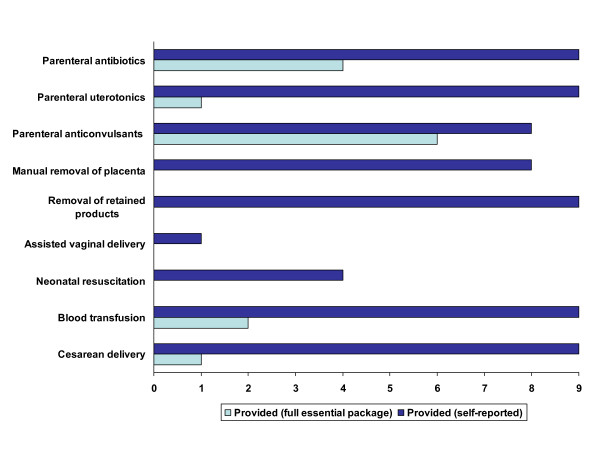
**Provision of EmOC signal functions through self-report or with full essential package at 9 hospitals in the DRC**. *Provided (self-reported) *defined as the facility staff reported in interviews that the facility had performed the signal function in the preceding 3 months. *Provided (full essential package) *defined as the facility had the complete package of supplies, equipment, drugs and staff to perform the signal function according to internationally recognized standards on the day of the assessment and facility staff reported in interviews that the facility had performed the signal function in the preceding 3 months.

Although none of the nine hospitals met the criteria for a functioning EmOC facility (either basic or comprehensive), all of the hospitals reported having provided the following signal functions in the preceding three months: administration of parenteral antibiotics and uterotonic drugs, removal of retained products of conception, blood transfusion and cesarean delivery (Figure 2). Only one hospital reported having performed an assisted vaginal delivery in the preceding three months, while four reported having performed neonatal resuscitation.

Aside from failing to perform all of the signal functions, the hospitals did not satisfy the criteria for functioning EmOC facilities primarily due to a lack of trained staff, limited availability of services (less than 24 hours a day) or lack of supplies and equipment for performing the signal functions to a quality standard. For example, no hospital possessed the complete package of essential supplies, equipment and staff to provide manual removal of the placenta, removal of retained products, assisted vaginal delivery or neonatal resuscitation.

Lack of medicines, supplies and equipment was a frequent barrier to the hospitals' provision of obstetric services. Of the five hospitals that lacked the full package to provide parenteral antibiotics and uterotonic drugs, three had no ampicillin, three had no oxytocin and four lacked needles and/or syringes. In addition, none of the hospitals was able to perform manual removal of the placenta because they lacked such basic supplies as dextrose or glucose as well as the RH-specific drugs oxytocin or ergometrine to aid the process. None of the hospitals had long gloves for use during the procedure.

Only one hospital was able to consistently and safely provide surgery for obstetric complications. The remaining hospitals did not have the appropriate and necessary equipment, surgical instruments, drugs or oxygen to safely perform a cesarean section.

Two of the nine hospitals possessed adequate equipment and supplies to provide safe blood transfusions. Of the seven remaining hospitals, five did not have a blood bank, four did not have airway needles and five did not have a functioning refrigerator. In addition, several hospitals did not have the kits to test transfused blood for Hepatitis B (one), Hepatitis C (three) or syphilis (two); all nine hospitals did, however, have HIV test kits.

Despite lacking equipment to provide cesareans or safe blood transfusions, all nine hospitals reported having performed these procedures in the three months prior to the assessment.

Lack of trained staff was documented as a major reason for not being able to perform the signal functions. Three hospitals were unable to perform assisted vaginal deliveries or neonatal resuscitation due to lack of training. Inconsistent provision of services, mainly at night or over weekends, was also an important reason for not providing the signal functions. While all of the hospitals had at least one medical doctor on staff, two hospitals had only one doctor and three had only two doctors. Four hospitals had fewer than ten nurses on staff (one hospital had only three nurses), four had more than ten nurses and one hospital did not report the number of nurses on staff. Four hospitals had three formally trained nurse-midwives, while one hospital had only one and four hospitals had none among their nursing staff. Only two hospitals had formally trained nurse-anesthetists; six had nurses who had been trained on the job to provide anesthesia; and one hospital had no one trained in anesthesia. Table 2 provides a summary of the relevant clinical staff at each hospital; clinicians at all government hospitals are assigned to their posts by the MOH.

**Table 2:** Clinical staff at 9 general referral hospitals, DRC

Province	Hospital	Medical doctors	Nurses	Nurse-midwives	Nurse-anesthetists	Nurses trained on-the-job in anesthesia
Kasai Occidental	Demba	2	2*	0	0	1
	
	Mutoto	1	3	0	0	2

Ndjili/Kinshasa	Roi Baudouin	9	39	0	0	2
	
	Kikimi	1	13	3	1	0
	
	HASC	39	47	1	7	0

Maniema	Kasongo	5	23	3	0	2

Orientale	Ubundu	4	6	3	0	0

South Kivu	Kabare	2	8	0	0	2
	
	Kalehe	2	7	3	0	7

*The number of all nurses at Demba Hospital was not recorded. This number represents only the number of senior level nurses.

### Family Planning

Family planning services were lacking in most of the hospitals assessed; only two hospitals provided a range of methods. Contraceptive implants were not offered at any of the hospitals; IUD and emergency contraception were offered at only one hospital; oral contraceptive pills and injectables were offered at two hospitals; and male and female condoms were offered at three hospitals. Six hospitals reported providing tubal ligation, but this was determined to be only when the procedure was recommended for medical reasons and performed during a cesarean; tubal ligation was not performed as an elective, scheduled FP procedure in any hospital. Of the two hospitals that provided multiple FP methods, only one had FP information (leaflets or a flipchart) available to assist with counseling. As with EmOC services, essential supplies such as needles, syringes or specula were missing from the outpatient room in which FP services were provided at both hospitals that offered FP services. One of these hospitals had no FP methods available in the outpatient room, although they were available in the pharmacy.

The main reasons reported for not providing FP methods were a lack of training for providing counseling to clients or for performing the procedures, closely followed by a lack of necessary supplies or equipment. Policy issues, related to religious beliefs, were cited by the Catholic hospital as the reason for not providing any FP services.

### Infection Prevention

Infection prevention practices were found to be inadequate in the delivery room, maternity ward and laboratory at all assessed facilities. Poor and nonexistent infection prevention practices were also documented in the operating theatre (seven) and outpatient areas (eight). The gaps in infection prevention were mainly due to the lack of equipment and supplies such as soap, washing stations, disinfectant solution, bleach, sharps containers or covered contaminated waste bins. Two hospitals had no functioning autoclave with which to sterilize equipment. Waste disposal was a notable issue: three hospitals had no incinerator, one had an incinerator too full to use and five hospitals did not separate clinical waste from other waste.

### Routine monitoring system

No facility had an adequate monitoring system for EmOC. Very few facilities recorded obstetric complications or the treatment provided in response, with the exception of cesarean sections. A lack of clear case definitions, such as for a complicated abortion, was also noted. Neither maternal deaths nor the causes thereof were clearly or consistently documented. In the two hospitals that offered FP, utilization data were more routinely recorded; however, neither facility had a system to track clients using short-term methods who defaulted.

## Discussion

The facility assessment results illustrate the serious gaps in existing RH services among general referral hospitals in the DRC and suggest areas where improvement can be made in order to make good quality RH services accessible to the population.

### General infrastructure

Issues of general infrastructure such as renovations to physical structures; re-organization of client flow through the facility; and installation or improvement of power, water, sanitation and waste management systems must be addressed to facilitate effective infection prevention and the provision of good quality RH services [27]. It is imperative for all facilities to maintain an adequate and sufficient water supply and to have clean water available inside the hospital. All hospitals must have at least two sources of electrical power, to ensure that power is available at all times without (or with minimal) interruption. Both primary and backup power systems require regular maintenance so that power outages are avoided.

### Commodities management

Commodity security and management were clear gaps that were identified in all of the facilities. At the time of the facility assessments, most facilities lacked the essential drugs, equipment and supplies, such as ampicillin, oxytocin, needles and syringes, needed to perform signal functions. Of the two hospitals that had provided short- and long-term FP methods in the prior three months, only one hospital had any FP counseling materials. Three hospitals indicated that despite having trained staff, they never stocked FP methods. Various reasons for not procuring FP supplies were mentioned by staff: they assumed women did not want FP or they feared the religious missions that managed the hospitals would prohibit the provision of FP.

The six hospitals with the essential package to provide parenteral anticonvulsants used diazepam, a less effective treatment for eclampsia [39]. Magnesium sulfate, the simplest and most effective treatment of eclampsia, was available in only one hospital. Updated RH protocols and essential drugs lists must reflect the most modern and effective drugs, equipment and procedures; these drugs and equipment must be available to procure in the country; and staff must be trained to ensure their appropriate use.

Interventions are limited if effective and reliable medical supplies and equipment are unavailable. The lack of these inexpensive, basic supplies demonstrates the need for systems to manage drug and supply chains. Insufficient support and poorly functioning systems during years of war mean few or no staff have the skills to properly manage a supply system. Training for hospital managers and medical personnel on drug and equipment procurement and management must be prioritized.

### Staffing

Implicit in the definition of an EmOC facility is that the signal functions be available to women 24 hours a day and seven days a week since demand for EmOC services cannot be predicted. The primary obstacle to the 24 hour provision of EmOC in the facilities studied was the lack of the essential health workers at the facility. According to the DRC's national protocol, a general referral hospital with 100 beds serving a population of 100,000 should have at least three doctors, one anesthetist and 16 nurses [40]. The majority of the hospitals assessed had fewer than this minimum. Unsurprisingly, the hospitals located in more remote and isolated areas had fewer staff than those in more urban or accessible areas. In many cases, the health zone medical officer (*Médecin chef de zone*) was counted as a doctor at the hospital despite his other non-clinical duties. The lack of a doctor is of particular importance with regards to procedures, such as cesarean deliveries, that only a doctor is authorized to perform. In some facilities, nurses were unofficially trained to perform cesareans. The researchers were unable to determine which procedures each level of provider was authorized to perform having received inconsistent responses from different MOH officials. Supporting the hiring and retention of skilled health workers at the facility (through provision of adequate housing and regular payment of salaries) and reviewing policies to expand the scope of services performed by non-physician clinicians would help improve 24 hour availability of EmOC [13,33] and make a broader range of FP methods available at health facilities.

### Training

Competency-based clinical training and continuing education are crucial to enable the health system to provide good quality care. In the nine hospitals assessed, lack of training was a barrier to the provision of both FP and EmOC services and was consistently ranked as the main reason that facilities did not provide RH services. For example, none of the nine hospitals was able to perform an assisted vaginal delivery due in part to lack of training. Conversations with Congolese physicians suggest that this signal function was often de-emphasized in physician training. Continuing education to update health workers on new more effective technologies was lacking as most facilities used outdated procedures and/or drugs. For example, all of the facilities performed dilation and curettage instead of manual vacuum aspiration (MVA), the relatively simple and safe alternative recommended by the WHO, for removal of retained products of conception.

Clinical training, including refresher training, should take into account both RH-specific and health systems approaches. An RH approach to training would provide hospital staff currently providing RH services with procedure-specific up-to-date in-service training. A health systems response to training would include a review of basic medical, nursing and midwifery training curricula to ensure the incorporation of appropriate training for the provision of FP methods, drugs and procedures to treat obstetric complications and infection prevention policies [41]. IRC is creating training centers at five supported hospitals to enable the trained staff to train clinicians from health centers in the health zone.

### Infection prevention

Although infection prevention practices at all of the hospitals were inadequate, this is an area in which low cost, low technology interventions can make a difference. Infection prevention policies and procedures are effective and relatively simple to implement. It is essential that all facility staff, whether they provide clinical care or not, be trained in good infection prevention practices and that the necessary equipment and supplies, such as incinerators and sharps containers, be available so that infection prevention policies and procedures can be followed. Even where EmOC or FP services are available, failure to follow infection prevention procedures can put both staff and patients at unnecessary risk and result in poor clinical outcomes.

### Policies and protocols

The availability and delivery of RH services are affected by national health policies and protocols; the omission of newer, safer and easier to use drugs and procedures from the DRC's RH policies and protocols has affected the availability of RH services at the studied facilities. Misoprostol, for example, an effective, inexpensive and easy-to-administer drug which can be used to prevent post-partum hemorrhage [42], is not included in the DRC's national RH norms. Although MVA does appear in the national RH norms [43], it is not consistently referenced in national RH policy documents. Further, MVA is found only in the norms for hospitals but not for health centers despite evidence that MVA can be safely provided at the health center level and performed by non-physician clinicians [44,45].

Even when updated RH policies were in place, some discrepancies between policy and practice were noted. Although some new drugs or procedures have been included in recent revisions of national health protocols, the lack of training or failure to procure the necessary drugs and equipment prevented their use. For example, magnesium sulfate, which is on the essential drugs list, was available in only one of the nine hospitals assessed. Non-governmental organizations (NGOs) working in the DRC have reported difficulty in identifying a local source for procurement. Likewise, differences were noted between the standard equipment for general referral hospitals designated by national policy and what was actually observed in the hospitals assessed. For example, vacuum extractors and MVA kits were included in the standard equipment list for a hospital in the DRC, yet most of the hospitals did not have this equipment [15]. In addition, neither appeared in the MOH definitive list of RH commodities to be secured [46]. Reasons for these discrepancies are not known, but could include the lack of effective equipment management and planning by the hospital or MOH or the active discouragement of the use of the procedures (for example, by encouraging the use of cesarean over assisted vaginal delivery). As noted previously, even if these equipment were available, staff in half of the hospitals lacked training to use them. It is imperative not only for updated and more effective drugs and procedures to be consistently included in national policies but also for the MOH to facilitate their use and implementation in health facilities through training and procurement.

### Referral systems

Effective, functioning referral systems are critical to the accessibility of RH services. All of the hospitals assessed are local referral hospitals for EmOC, yet travel to these hospitals may not be feasible for women experiencing obstetric complications because of distance, cost of transportation or poor road infrastructure. The lack of ambulances is a serious problem throughout the DRC; however, in some of the rural areas where these hospitals are located, roads are impassable to four-wheel vehicles during rainy season. Where transport is extremely difficult, CARE is ensuring that basic EmOC is available in health centers that are furthest and least accessible to the hospital. Alternative transportation options, such as motorcycle ambulances, bicycle taxis and commercial vehicles should be explored. Community savings groups, community insurance and income generation activities are all approaches that might be used to assist women and their families to pay for these critical services [47].

### Information systems, monitoring and evaluation

A key feature of a sustainable and functioning health infrastructure is the assessment, monitoring and evaluation of services [33]. The UN Process Indicators have been shown effective tools to guide the design of EmOC programs and to monitor the provision of EmOC services [13,48]. In the nine hospitals assessed, monitoring of EmOC was virtually nonexistent. The obstetric registers were so poor that it was difficult to determine reliable baseline levels for some of the UN Process Indicators. Monitoring performance allows facility staff to better understand which service areas are not functioning and the reasons why so that they can initiate improvements. At the facility level, all staff should receive training, regular supervision and support in maintaining and using monitoring systems.

Obstetric registers should be revised so that key data are included and less important data are excluded. Standard case definitions should be shared with all staff working in the maternity; the staff must understand the importance of collecting good quality data and how to use these data. Monitoring of services can help the facility management better understand patient flow and volume, which has implications for needed program inputs [10]. Furthermore, consistent monitoring using the UN Process Indicators has proven to be an effective way to assess maternal mortality reduction and improve the functioning of EmOC facilities [49]. In addition, facilities may wish to collect other information to gain more insight into the quality of care including the time elapsed between a woman's admission to an EmOC facility and her actual receipt of treatment, and detailed case reviews of both maternal deaths and 'near misses' (i.e., women who experience an obstetric complication, are treated in the facility and survive) [13,50].

### Study Limitations

The facility assessment only provides a snapshot of staff, services, equipment and supplies that were available and functioning on the day of the assessment and cannot evaluate those that were available at any other period of time. It is feasible that a hospital may have been able to provide certain services in the past but was classified as not being capable of doing so due to the lack of essential drugs, equipment, staff or supplies on the day of the assessment. In addition, this assessment did not explore the quality of the services provided by individual health workers. Despite these limitations, it is clear that major improvements are needed at all of the hospitals assessed.

## Conclusions

Access to RH care is a basic human right, yet integrated and fully comprehensive RH services based on sound facility assessment data are not the norm in most emergency and post-emergency settings. In the DRC, preventable deaths and illnesses related to RH are all too common and women and men are routinely denied their right to health including RH. Women's lives can be saved and their well-being improved with functioning RH services. Inexpensive and effective interventions are available to prevent unintended pregnancy, help women safely through pregnancy and childbirth and prevent infections in the hospital setting [3]. None of these interventions, however, can work without a functioning health system in place. There is a growing consensus that building stronger health systems is key to achieving improved health outcomes, especially in countries where the health indicators are the worst [41], and may contribute to state building and peace promotion in post-conflict countries [51]. Although not normally in the mandate of humanitarian NGOs, supporting the MOH and building local capacity to revitalize the health system is imperative in post-conflict or long-term stabilized conflict settings. Post-conflict reconstruction efforts in the DRC must focus on improving the health infrastructure and ensuring the availability of RH services if avoidable maternal deaths are to be eliminated.

As it emerges from over a decade of war, the DRC has the opportunity and obligation to make progress towards achieving the MDG 5 and to improve RH service delivery. IRC and CARE are working with RAISE, the local MOH and other partners to 1) support the improvement of the basic health infrastructure by providing necessary equipment and renovations for EmOC to general referral hospitals, 2) assist facilities and health zone offices with supplies management, 3) provide comprehensive competency-based training for health providers in EmOC, FP and infection prevention, 4) improve the referral system to the general referral hospitals, 5) advocate for changes in national RH policies and protocols and participate in national-level policy review processes and 6) provide technical assistance for monitoring and evaluation of key RH indicators.

Despite the inherent challenges of working in complex humanitarian situations, women and men in conflict-affected countries have the same rights to RH as those living in non-conflict settings. Together, these initiatives will improve the quality and accessibility of RH services in the DRC - services which are urgently needed and to which Congolese women and men are entitled by international human rights law.

## Competing interests

The authors declare that they have no competing interests.

## Authors' contributions

SC and JA conceptualized and designed the assessment; SC provided training and technical assistance to field teams; KM, IMA, MMH, BA and PK supervised data collection; KM, IMA, MMH, BA and PK performed preliminary data analysis; SC evaluated the data; SC conceptualized the paper and was principle author; KM & JA contributed to the writing process; all authors reviewed the final text.
